# Mandible-derived extracellular vesicles regulate early tooth development in miniature swine via targeting KDM2B

**DOI:** 10.1038/s41368-025-00348-w

**Published:** 2025-04-27

**Authors:** Ye Li, Meng Sun, Yi Ding, Ang Li

**Affiliations:** 1 Key Laboratory of Shaanxi Province for Craniofacial Precision Medicine Research, College of Stomatology, Xi’an Jiaotong University, Xi’an, China; 2 Department of Periodontology, College of Stomatology, Xi’an Jiaotong University, Xi’an, China

**Keywords:** Developmental biology, Embryonic germ cells, Cell biology

## Abstract

Tissue interactions play a crucial role in tooth development. Notably, extracellular vesicle-mediated interactions between the mandible and tooth germ are considered essential. Here, we revealed that mandible extracellular vesicles could modulate the proliferation and differentiation of dental mesenchymal cells by regulating the histone demethylase KDM2B. Further investigation showed that mandible derived extracellular vesicles could deliver miR-206 to KDM2B, thereby regulating tooth development. An animal study demonstrated that the miR-206/KDM2B pathway affected tooth morphogenesis and mineralization after eight weeks of subcutaneous transplantation in nude mice. In conclusion, this study suggested that the mandible played a critical role in tooth morphogenesis and mineralization, which could be a potential therapeutic target for abnormal tooth development and an alternative model for tooth regeneration.

## Introduction

Tooth loss is increasingly recognized as a global issue, causing significant psychological and physical distress to individuals and imposing substantial social and economic burdens.^1,2^ In contrast to traditional restoration treatments, which lack biological functionality, tooth regeneration presents distinctive advantages and is emerging as a prominent trend driven by the desire to achieve biological and physiological regrowth of lost teeth.^3,4^ Through the emulation of natural tooth formation processes in vivo, cell-tissue recombination emerges as a promising avenue for crafting functional substitutes for lost teeth.^5,6^ Nonetheless, the journey towards de novo odontogenesis has encountered notable disparities compared to natural tooth development, despite various regenerative endeavors.

A comprehensive grasp of the molecular mechanisms governing natural tooth development is essential for devising effective strategies for tooth regeneration.^7^ The intricate nature of tooth development arises from the intricate interplay among various tissues. Particularly, the reciprocal interactions between epithelial and mesenchymal tissues are pivotal for orchestrating biological processes, such as tooth formation.^8–11^ However, despite its close functional and positional ties to the tooth, mandibular functions in tooth development have been relatively underexplored.^12^ Our previous investigation demonstrated the involvement of extracellular vesicles released from the mandible (mandible-EVs) in regulating tooth development, underscoring the critical importance of communication between teeth and the mandible in maintaining normal tooth development.^13^ However, the underlying mechanism by which mandible regulates tooth development remains to be elucidated.

Epigenetic modifications, including non-coding RNA regulation and histone modification, play pivotal roles in governing tooth development.^14,15^ Methylation of lysine residues on histones constitutes a crucial aspect of epigenetics and significantly influences tooth development.^16^ The spatial and temporal expression patterns of H3K4me3 and H3K27me3 are modulated by methyltransferases such as SET7 and EZH2, as well as demethylases like KDM5B and JMJD3, throughout murine tooth germ development.^17^ These alterations hold profound significance in regulating cellular differentiation during tooth development processes. Additionally, KDM5A-mediated histone demethylation has been shown to regulate the osteogenic differentiation of human dental pulp stem cells, thereby impacting tooth development and reparative dentinogenesis.^18^ Furthermore, non-coding RNAs have been implicated in modulating histone methylation, adding further complexity to their influence on tooth development.^19^ For instance, miR-140-3p has been reported to regulate osteo/dentinogenic differentiation of dental pulp stem cells by targeting KMT5B.^20^ Nevertheless, whether epigenetic modifications contribute to mandible-tooth signaling during tooth morphogenesis remains an area requiring further elucidation.

In this study, we investigated the molecular mechanisms of mandible-tooth interaction to gain insight into tooth development. We have revealed that the mandible is capable of secreting extracellular vesicles, thereby regulating tooth development through epigenetic mechanisms. Our findings shed light on the regulatory role of the mandible in tooth formation, potentially offering insights into preventing and treating abnormalities in tooth development, as well as presenting innovative strategies for tooth regeneration.

## Results

### The mandible regulated the early tooth development through extracellular vesicles

**Fig. 1:**
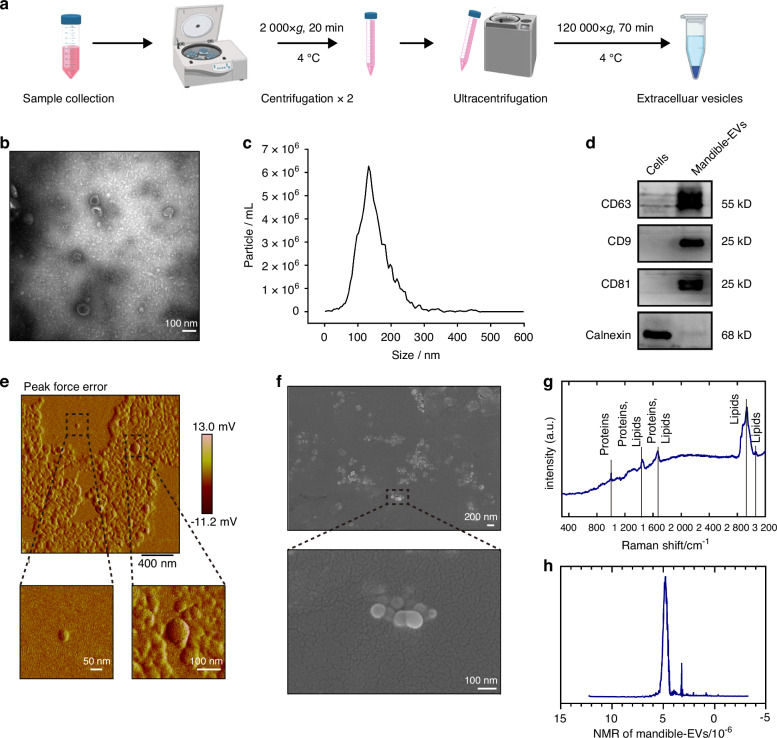
Extraction and identification of extracellular vesicles. **a** Schematic diagram of the extracellular-vesicle extraction process. **b** Transmission electron micrographs of mandible-EVs. Scale bar: 100 nm. **c** Particle sizes measured by nanoparticle tracking analysis. **d** Western blotting characterization of EV markers. **e** Topography of mandible-EVs detected by AFM. **f** Topography of mandible-EVs detected by SEM. **g** Raman spectra of mandible-EVs. **h** Representative 600 MHz 1H NMR of mandible-EVs. Sample, conditioned media collected after culture of miniature pig mandible with tooth germ tissue removed

**Fig. 2:**
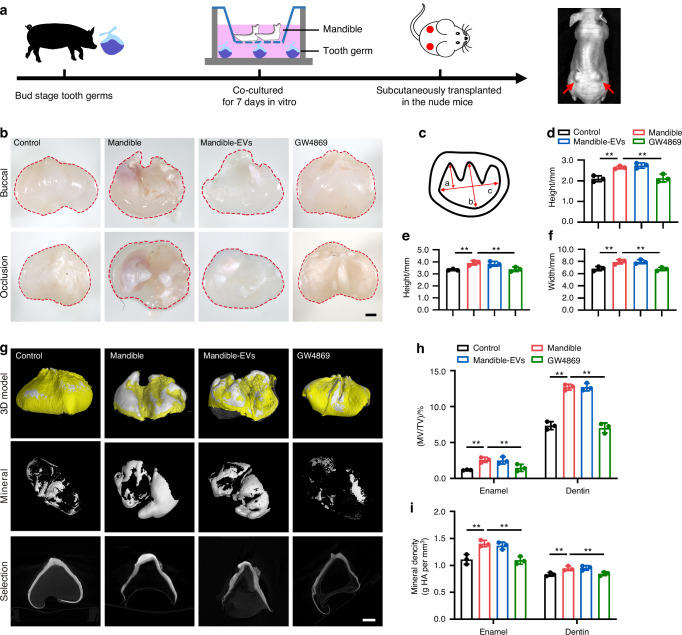
Mandible regulated the early tooth development through extracellular vesicles. **a** Schematic illustration of the experimental procedure. **b** Morphological characteristics under a stereomicroscope. Scale bar: 1 mm. **c** Schematic of the method for measuring tooth morphology. **d** The cusp height of tooth. **e** The height of tooth. **f** The width of tooth. **g** Three-dimensional reconstruction of micro-CT images. Scale bar: 1 mm. **h**, **i** Quantification analyses of MV/TV, and Mineral density. MV/TV, mineral volume/tissue volume; The enamel and dentin density ranges were defined as (1.25 ± 0.10) g/cm^3^ and (0.89 ± 0.05) g/cm^3^; ** *P* < 0.01; *n* = 3

To clarify the function of mandible regulation in tooth development, we co-cultured minipig premolars at E40 (which is bud stage of tooth development deciding the shape and mineralization of the tooth phenotype) with minipig mandible or mandible-EVs in a Transwell system after EV characterization (Fig. 1b-d). To gain a comprehensive understanding of the physical properties and chemical composition of mandible-EVs atomic force microscopy (AFM), scanning electron microscopy (SEM), Raman spectroscopy and nuclear magnetic resonance (NMR) were employed. The mandible-EVs typically appeared as round vesicles with a size of about 50-150 nm and a rough surface (Fig. 1e, f). Raman spectroscopy and NMR results showed that the chemical composition of mandible-EVs mainly included lipids and proteins (Fig. 1g, h). Then tooth germs were transplanted subcutaneously into nude mice to assess tooth development in vivo (Fig. 2a). Eight weeks after transplantation, the tooth tissues were harvested. As expected, developed premolars were detected in the co-cultured group. However, the teeth treated by GW4869 (which is commonly used as inhibitor of extracellular vesicle secretion) were smaller and had different morphological abnormalities compared to teeth in control group (Fig. 2b-f). Further micro-CT results revealed that, in addition to the impaired morphology, the mineralized tissue was significantly reduced in the extracellular vesicle inhibition group (Fig. 2g–i). HE staining results for each group were given in Fig. S1. We found that mandible-EVs regulated tooth morphogenesis and mineralization during early tooth development.

### The mandible-EVs inhibited proliferation and promoted odontogenic differentiation of dental mesenchymal cells at E40

**Fig. 3:**
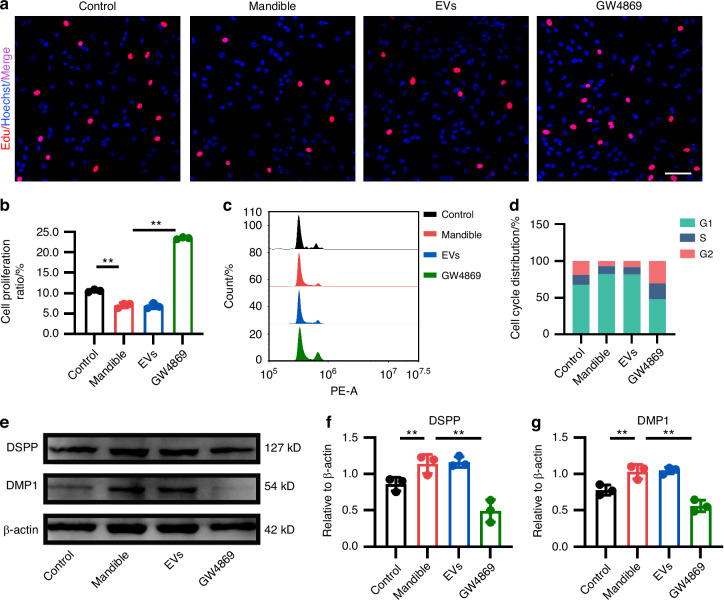
Mandible-EVs inhibited proliferation and promoted odontogenic differentiation of dental mesenchymal cells at E40. **a** EdU assays were applied to confirm the proliferation ability of dental mesenchymal cells. Scale bar: 200 μm. **b** Quantitative analysis. **c** To identify the effect of mandible/ mandible-EVs on cell cycle distribution, flow cytometry (FCM) was performed. **d** Percentage of G1, S, and G2 cell populations in the cycle phase based on FCM. **e** The protein level of DSPP and DMP1. **f**, **g** Western Blotting quantification. n.s not significant, * *P* < 0.05, ** *P* < 0.01

To further clarify how mandible-EVs regulate early tooth development, we investigated the effect of mandible-EVs on dental mesenchymal cell fate decisions. The EDU assay was employed to investigate the impact of mandible-EVs on cellular proliferation. The findings indicated that mandible-EVs had a strong suppressive effect on the proliferation of dental mesenchymal cells (Fig. 3a, b). Flow cytometry also indicated that mandible-EVs significantly inhibited the proliferation of dental mesenchymal cells (Fig. 3c, d). In addition, we verified the expression levels of the odontogenic differentiation markers *DSPP* and *DMP1* using RT-qPCR (Fig. S3). Western blotting was employed for further validation (Fig. 3e-g). The results showed that mandible-EVs could promote the odontogenic differentiation of dental mesenchymal cells.

### KDM2B was the downstream target of mandible-EVs during tooth development

**Fig. 4:**
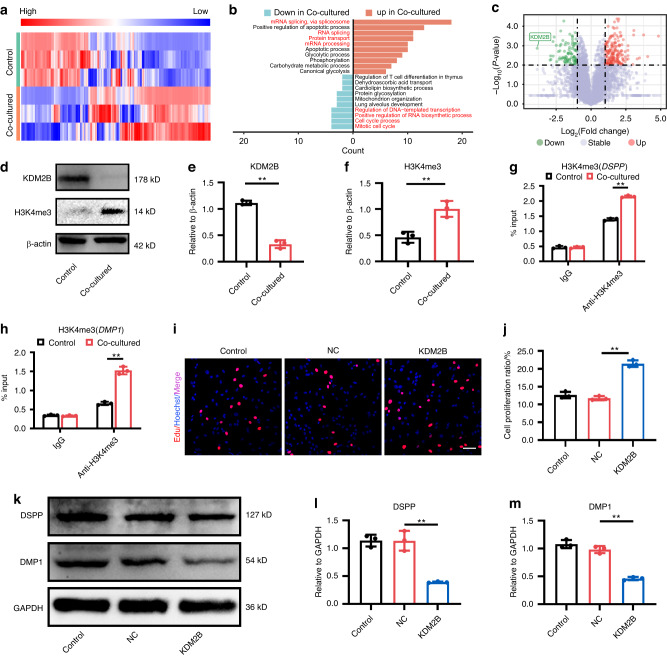
KDM2B was the downstream target of mandible-EVs during tooth development. **a** Heatmap of differentially expressed proteins in tooth germs co-cultured with mandible-EVs. **b** Gene Ontology (GO) analysis of proteins differentially expressed from tooth germs co-cultured with mandible-EVs. **c** Volcano plot filtering of differentially expressed proteins. **d** The level of KDM2B and H3K4me3 in dental mesenchymal cells co-cultured with mandible-EVs. **e**, **f** Western blotting quantification. **g** The enrichment of H3K4me3 at the promoter region of *DSPP* regulated by mandible-EVs. **h** The enrichment of H3K4me3 at the promoter region of *DMP1* regulated by mandible-EVs. **i** EdU assays were applied to confirm the proliferation ability of dental mesenchymal cells. Scale bar: 200 μm. **j** Quantitative analysis. **k** The protein level of DSPP and DMP1 in dental mesenchymal cells treated with KDM2B. **l**, **m** Western blotting quantification. n.s not significant, * *P* < 0.05, ** *P* < 0.01

To demonstrate the critical role of mandible-EVs in early tooth development, mass spectrometry was performed (Fig. 4a). Extracellular vesicles from the E40 mandible were retrieved and co-cultured with tooth germs. As shown in Fig. 4b, proteins involved in cell differentiation were preferentially upregulated when co-cultured with mandible-EVs. While proteins associated with cell proliferation were expressively downregulated. Results in Fig. 4c demonstrated that histone demethylase KDM2B was the most significantly downregulated after co-culture. The expression of KDM2B levels was further validated by Western blotting. KDM2B was suppressed in co-cultured dental mesenchymal cells, whereas its target H3K4me3 was significantly increased (Fig. 4d-f). It was further indicated that mandible-EVs promoted the enrichment of H3K4me3 at the promoter regions of *DSPP* and *DMP1* (Fig. 4g, h).

To demonstrate the critical role of KDM2B in tooth development, we overexpressed KDM2B in dental mesenchymal cells (Fig. S4a, b). The EDU experiment showed that KDM2B overexpression correlated significantly promoted the cell abilities to proliferate (Fig. 4i, j). Flow cytometry and CCK8 were employed to further clarify the proliferation of dental mesenchymal cells (Fig. S4c-e). As shown in Fig. S4f-g and Fig. 4k-m, the expression of dentinogenic markers DSPP and DMP1 were decreased when KDM2B was overexpressed. Above all, KDM2B played a critical role in the regulation of dental mesenchymal cell cellular events.

### The regulation of KDM2B on dental mesenchymal cell proliferation and differentiation was reversed by miR-206

**Fig. 5:**
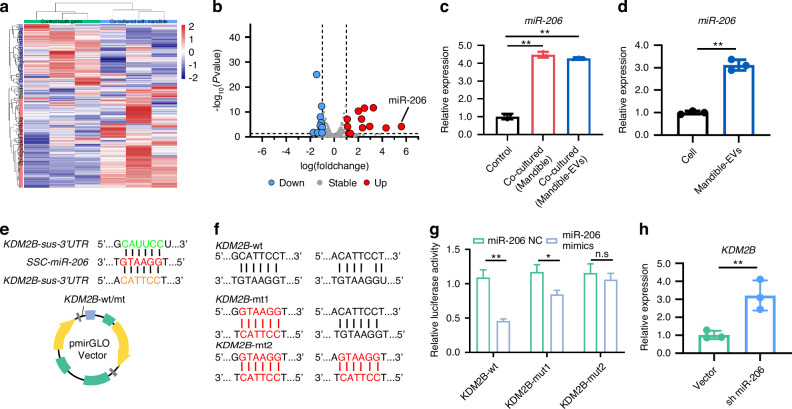
KDM2B was the downstream target of miR-206. **a** Heatmap of differentially expressed miRNAs in tooth germ co-cultured with mandible-EVs. **b** Volcano plot filtering of differentially expressed miRNAs. **c** The level of miR-206 in co-cultured dental mesenchymal cells. **d** The level of miR-206 in mandible-EVs. **e**, **f** Luciferase reporter designing. **g** Transfection of miR-206 mimics with luciferase reporter. **h** The mRNA level of KDM2B regulated by miR-206. n.s not significant, * *P* < 0.05, ** *P* < 0.01

To gain further insight into KDM2B in regulating tooth development, we employed a small RNA sequence to identify the potential upstream regulatory molecule of KDM2B (Fig. 5a). MiR-206 was selected as a putative effector due to its markedly increased expression in mandible-EVs co-cultured tooth germs (Fig. 5b). We further quantitated the expression of miR-206 in dental mesenchymal cells cultivated alone and with mandible-EVs. Compared with the control group, the expression of miR-206 was significantly increased in co-cultured dental mesenchymal stem cells (Fig. 5c). We further detected the level of miR-206 in mandible-EVs (Fig. 5d). As shown in Fig. 5e, KDM2B had two putative miR-206 binding sites in its 3’- UTR, according to which we constructed the wild-type or mutant plasmid of KDM2B and co-transfected into 293 T cells (Fig. 5f). According to the luciferase reporter assay, miR-206 mimics resulted in the inhibition of luciferase activity, indicating the direct binding of miR-206 to KDM2B (Fig. 5g). To further validate the effects of miR-206 on KDM2B, we then tested the expression of KDM2B in response to the suppression of miR-206. As expected, the mRNA of KDM2B was significantly up-regulated by miR-206 inhibition (Fig. 5h). Taken together, these findings indicated the direct binding of miR-206 to KDM2B.

**Fig. 6:**
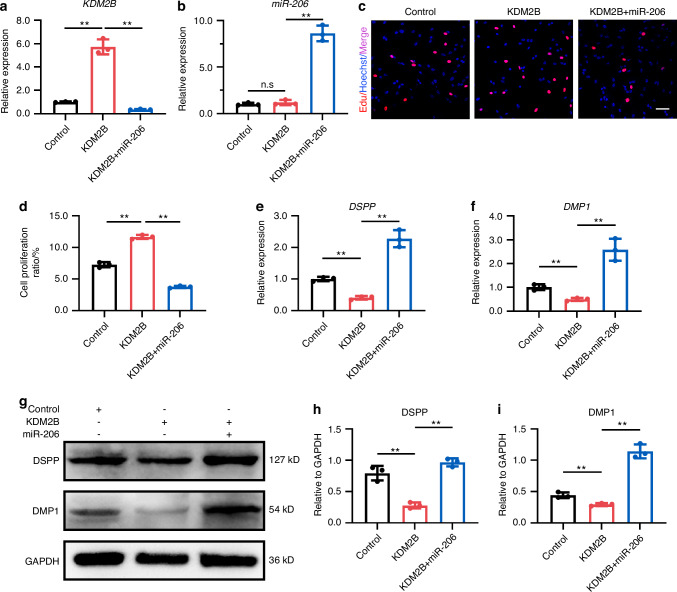
Mandible-EVs transferred miR-206 to regulate cell proliferation and differentiation of dental mesenchymal cells. **a** The mRNA level of *KDM2B* in dental mesenchymal cells treated by KDM2B or miR-206/KDM2B. **b** The level of *miR-206* in dental mesenchymal cells treated by KDM2B or miR-206/KDM2B. **c** EdU assays were applied to confirm the proliferation ability of dental mesenchymal cells regulated by miR-206/KDM2B. Scale bar: 200 μm. **d** Quantitative analysis. **e** The mRNA level of *DSPP* regulated by miR-206/KDM2B. **f** The mRNA level of *DMP1* regulated by miR-206/KDM2B. **g** The protein level of DSPP and DMP1 regulated by miR-206/ KDM2B. **h**, **i** Western blotting quantification. n.s not significant, * *P* < 0.05, ** *P* < 0.01

We further explored the signaling pathway modulated by miR-206/KDM2B in dental mesenchymal cell proliferation and differentiation. The overexpression of KDM2B and simultaneous upregulation of miR-206 were performed in dental mesenchymal cells (Fig. 6a, b, Fig. S5a). As expected, KDM2B promoted dental mesenchymal cell proliferation, and miR-206 significantly reversed the effect of KDM2B on dental mesenchymal cell proliferation (Fig. 6c, d, Fig. S5b-d). As indicated in Fig. 6e-i, the level of DSPP and DMP1 was significantly reduced in KDM2B treated cells and restored in cells co-transfected with the KDM2B and miR-206.

### MiR-206/KDM2B regulated early tooth development by modifying dental mesenchymal cell proliferation and differentiation in vivo

**Fig. 7:**
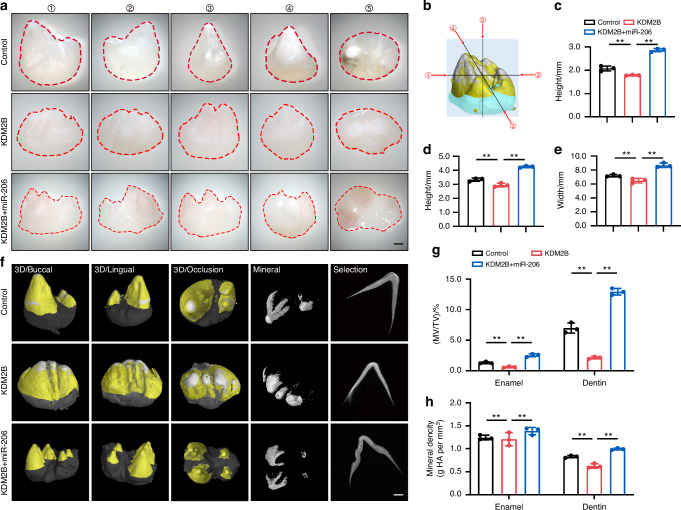
MiR-206/KDM2B regulated early tooth development in vivo. **a** Morphological characteristics under a stereomicroscope. Scale bar: 1 mm. **b** Schematic of the method for measuring tooth morphology. **c** The cusp height of tooth. **d** The height of tooth. **e** The width of tooth. **f** Three-dimensional reconstruction of micro-CT images. Scale bar: 1 mm. **g**, **h** Quantification analyses of MV/TV, and Mineral density. MV/TV, mineral volume/tissue volume; The enamel and dentin density ranges were defined as (1.19 ± 0.10) g/cm^3^ and (0.68 ± 0.09) g/cm^3^; ** *P* < 0.01; *n* = 3

To elucidate the in vivo effect of miR-206/KDM2B on tooth development, we transfected a lentiviral vector overexpressing KDM2B into E40 miniature swine premolars with or without miR-206 rescue (Fig. S6a). The development of tooth germs was examined 8 weeks after subcutaneous transplantation in nude mice. To our expectation, KDM2B overexpression impacted tooth morphogenesis and mineralization, particularly cusp formation, while miR-206/KDM2B premolars were well-developed (Fig. 7a–e). The result of micro-CT indicated a lower number of cusps and a marked decrease in mineralized tissue in the KDM2B-treated group (Fig. 7f). In contrast, the group treated with miR-206/KDM2B showed a significant increase in both mineralized tissue volume and density compared to the control group (Fig. 7f-h). HE staining results are shown in Fig. S6b. As shown in Fig. S6b-e, expression of DSPP and DMP1 decreased in the KDM2B treated group as expected, while the up-regulated of miR-206 was capable of reversing the negative effect.

## Discussion

In our recent study, we illustrated how mandible-derived extracellular vesicles (mandible-EVs) fostered tooth morphogenesis and mineralization both in vitro and in vivo. Crucially, this phenomenon was substantially modulated by epigenetic mechanisms facilitated by miR-206 and its target gene, KDM2B. The miR-206/KDM2B axis effectively governed mesenchymal cell proliferation and odontogenic differentiation throughout tooth development.

Tissue interactions play a pivotal role in the normal growth of teeth.^21,22^ Despite the well-established connection between the mandible and tooth development, the specific role of the mandible and the underlying mechanisms remain poorly understood.^23,24^ Studies by Alfaqeeh et al. have suggested that tooth germ volume increases when the tooth is separated from the alveolar bone.^25^ Elodie’s observations highlighted the necessity of three-dimensional support for cusp pattern formation.^26^ While these studies imply the involvement of the mandible in tooth development, the precise mechanisms remain unclear. In our present study, we aimed to elucidate the function of the mandible during tooth development and unravel the mechanisms by which it regulates tooth size and morphology. We found that the mandible achieves this regulation by releasing extracellular vesicles that target tooth germ.

According to the findings of this study, mandible-EVs play a regulatory role in tooth development. Indeed, various signaling modalities, including the direct release of active chemicals by cells, contribute to the regulation of tooth development.^27,28^ These signals are transmitted and received to modulate internal processes. One crucial signaling involved in tooth development is parathyroid hormone-related protein (PTHrP), which acts as a paracrine and/or autocrine regulator of cell proliferation, apoptosis, and differentiation.^29^ Studies have shown that deletion of the PTHrP receptor in the dental follicle results in tooth eruption failure and shortened roots lacking periodontal ligaments.^30^ The utilization of extracellular vesicles for intercellular communication offers the potential to transfer unstable molecules such as miRNAs across cells.^31^ For instance, Jiang N et al. demonstrated that extracellular vesicles may transfer miR-135a, thereby inducing the transformation of epithelial/mesenchymal cells into enamel/dentin-forming cells by targeting the Wnt signaling pathway.^32^ In our current investigation, we observed that KDM2B expression was down-regulated in tooth germs co-cultured with mandible-EVs. Furthermore, the regulatory effect of miR-206/KDM2B in tooth germs was significantly suppressed when mandible-EVs were inhibited by GW4869. These findings suggest that the interactions mediated by extracellular vesicles between the mandible and tooth germs are crucial for regulating tooth development.

In our animal experiments, we observed that up-regulation of KDM2B expression severely hindered tooth morphogenesis and mineralization. Tooth agenesis presents a significant clinical challenge due to the multifaceted nature of its causes and manifestations.^33,34^ Mutations in the Msx1 gene have been implicated in halting tooth morphogenesis at the bud stage, indicating its potential role as a pathogenic gene for tooth agenesis.^35^ Loss-of-function mutations in genes such as PAX9 and WNT10A have also been associated with familial tooth agenesis (FTA).^36^ In our current investigation, we observed that miR-206 effectively reversed dental developmental abnormalities resulting from KDM2B upregulation. This discovery unveils a novel mechanism and potential therapeutic target for addressing tooth agenesis. Furthermore, extracellular vesicles offer a distinctive delivery approach for therapeutic interventions, owing to their ability to precisely target receptor cells.

Understanding the intricate mechanisms governing tooth development significantly contributes to the progress of tooth regeneration efforts.^37,38^ For instance, induction of BMP-4 in induced neural crest-like cells led to their differentiation into odontoblast-like cells expressing DMP1 and DSPP.^39^ When transplanted, these induced neural crest-like cells gave rise to well-organized and vascularized pulp-dentin complexes in vivo. However, attempts to generate tooth-like material through recombinant odontogenic epithelium and mesenchymal tissue have resulted in structures with notable size and morphological disparities compared to normal teeth.^40,41^ This difference is likely attributed to the absence of crucial developmental signals necessary for proper tooth formation.^42^ Exploring the impact of the mandible on tooth growth is crucial for enhancing the outcomes of tooth regeneration efforts. By uncovering these mechanisms, we set the stage for developing more efficient strategies in tooth regeneration and tissue engineering.

## Conclusions

In summary, our findings revealed that mandible-EVs facilitated early tooth morphogenesis and mineralization by transporting miR-206. Through its interaction with KDM2B, miR-206 inhibited the proliferation of dental mesenchymal cells while promoting their odontogenic differentiation, thus contributing to early tooth morphogenesis and mineralization.

## Materials and methods

### Extracellular vesicle isolation and characterization

The mandible and tooth germ tissues were isolated from miniature pigs at E40. The mandible tissue from 40 days embryonic development miniature pig is about 2 cm. After 1 day of adaptive culture in vitro, exosomes-free medium was used to culture the mandible. Then the medium was collected 3 days. The protocols for isolation and characterization were performed as previously described.^43^ Briefly, the Mandible culture medium was centrifuged twice at 2 000 × *g* for 20 min to remove cells and cell debris. The supernatant was collected and further centrifuged at 120 000 *g* at 4 °C for 70 min twice. Mandible-EVs precipitate was resuspended in cooled PBS. Then transmission electron microscopy (TEM) was utilized to identify morphology, nanoparticle tracking analysis (NTA) was performed to determine the size and concentration, and western blotting was employed to identify extracellular vesicle-specific markers.

### Transwell system

The tooth germ tissues or dental mesenchymal cells were cultured in exosomes-free medium in the lower chamber of the six-well plate Transwell system (0.4 μm, 723101, Nest, China), while the mandible was in the upper chamber.

### Animal experiment arrangement

Molar tooth germ, mandible and mandible-EVs were separated from 40 days embryonic development miniature pigs.

For the control group: the tooth germ was not specially treated; for the Mandible group: the tooth germ was co-cultured with mandible in a Transwell system; for the Mandible-EVs group: the tooth germ was firstly cultured with extracellular vesicle-free medium and Mandible-EVs from miniature pigs were then added in the co-cultured Transwell system; for the GW4869 group: the tooth germ was co-cultured with mandible and GW4869 was added simultaneously; for the KDM2B group: the tooth germ was treated with KDM2B overexpressed adenovirus vector; for the miR-206 group: the tooth germ was treated with *miR-206* overexpressed lentiviral vector.

### Subcutaneous transplantation

The nude mice (5-week-old) were used for transplantation. E40 molars from miniature pigs of each group were subcutaneously transplanted into the back of nude mice. Ketamine was injected intraperitoneally at 100 mg/kg before surgery to ensure that no pain was felt. Isoflurane anesthesia was performed during the surgery. Mice were euthanized by overdosage of CO_2_ inhalation followed by decapitation. Micro-CT analysis and other measurements were performed 8 weeks after transplantation. A total of 11 nude mice and 11 miniature pig fetuses were used for the experiments. All animal experiments followed the protocols and policies approved by the animal care and use committee at the College of Stomatology, Xi’an Jiaotong University (Permit Number: XJTUAE-2014-1538). Our study has complied with the ARRIVE 2.0 (Animal Research: Reporting of In Vivo Experiments) guidelines.

### Micro-CT (μCT) analysis

For quantification of mineralized tissue, tooth germ was scanned by a micro-CT (AX-2000, Always Imaging, China). The voltage was 70 kV, the current was 140 μA, the resolution was 5.9 μm, the projection number was 1440, and the integration time was 500 ms. VG Studio MAX 3.5 software (Volume Graphics) was used to analysis and three-dimensional reconstruct. The grayscale value of (19 139.00–39 869.00) ± 2 639.766 was defined as dentin and (39 870.00-58 147.00) ± 1 897.041 was defined as enamel for quantifying the mineralization.

### EDU assay

Cell proliferation was detected using the BeyoClick EdU cell Proliferation Kit with Alexa Fluor 555 (Beyotime Biotechnology, Shanghai, China) according to the manufacturer’s instructions. Briefly, dental mesenchymal cells transfected with lentiviral vector were incubated with 10 μmol/L EdU for 2 h, fixed in 4% paraformaldehyde for 15 min, and subsequently photographed after staining with Hoechst.

### Flow cytometry

The treated dental mesenchymal cells were collected and washed with cold PBS. After centrifugation at 1 000× *g* for 5 min, the cells were fixed with cold 70% ethanol for 12 h at 4 °C. Then 500 μl staining buffer, 25 μL propidium iodide (PI) staining solution and 10 μL RNase A (Beyotime, C1052, China) was added and incubated in the dark for 30 min. Cell cycle distribution was assessed via flow cytometer (Agilent, NovoCyte, USA).

### Cell counting kit 8 assay

The treated dental mesenchymal cells were inoculated in 96-well plates at the density of 1 × 10^4^ cells per well. After 0, 24, 48, or 72 h of culture, 10 μL CCK-8 solution (Boster Biotechnology, AR1199, China) was added into each well and incubated in the dark at 37 °C for 2 h. The absorbance was measured using a microplate reader (BIOTEK, EPOCH2, USA) at 450 nm.

### Western blotting

The total protein was isolated using RIPA lysis buffer containing protease inhibitors (Beyotime, Shanghai, China). The western blotting was performed as previously described. The primary antibodies used in this study are as follows: Rabbit Anti-DSPP antibody (bs-10316R, Bioss, China), Rabbit Anti-DMP1 antibody (1:500, bs-12359R, Bioss, China), FBXL10 (D3T8J) Rabbit mAb (1:500, #44570, Cell Signaling Technology, USA), Tri-Methyl-Histone H3 (Lys4) (C42D8) Rabbit mAb (1:500, #9751, Cell Signaling Technology, USA).

### Mass spectrometry

E40 tooth germs were co-cultured with 50 μg/mL EVs derived from the mandible for 7 days. Both the control tooth germs and the co-cultured tooth germs were quick-frozen and the protein profiles were identified.

### ChIP-qPCR

Cells were fixed and crosslinked utilizing 1% formaldehyde incubated at 37 °C for 10 min. Cross-linking was blocked by glycine at room temperature for 5 min. Then the cells were washed twice with ice-cold PBS and incubated with lysis buffer for 20 min at 4 °C. The mixture was centrifugated at 2 000 r/min rpm for 5 min. The cell precipitates were resuspended using pre-cooled lysis buffer and ultrasonication was further performed ultrasonic crushed using VCX750, 25% power, 4.5 S shock, 9 S gap for 14 cycles. Then the mixture was again centrifuged at 10 000 × *g* for 10 min at 4 °C. Antibodies against H3K4me3 antibody were added and incubated overnight at 4 °C. After overnight incubation, 60 μl of Protein A agarose was added and incubated for 4 h at 4 °C. The beads were washed twice using a wash solution. Then the samples were resuspended by elution buffer by pipetting up and down and incubated for 4 h at 65°C. DNA was purified with DNA purification kit and further used for Quantitative real-time PCR (RT-qPCR) assay.

### Small RNA sequence

E40 tooth germs were co-cultured with 50 μg/mL EVs derived from the mandible for 7 days. Both the control tooth germs and the co-cultured tooth germs were collected in order to extract miRNA. Small RNA sequencing was conducted and subsequently analyzed (Personalbio, Shanghai, China).

### Luciferase reporter assay

For target validation, luciferase reporter assay was performed to examine the interaction between KDM2B and miR-206 according to the manufacturer’s instructions. Briefly, KDM2B 3’UTR‐MUT and KDM2B 3′UTR‐WT were inserted into pmir-glo vectors. The miR‐206 mimics along with the vector was co-transfected with firefly luciferase 3′‐UTRs (KDM2B‐WT or KDM2B‐MUT) into 293T cells by Lipofectamine®3000. 48 h after transfection, a dual‐luciferase enzyme reporter assay was adopted to measure luciferase enzyme activities.

### Immunofluorescence

Tooth germs were fixed in 4% paraformaldehyde at 4 °C for 24 h. After decalcified and embedded the tissues were sliced into sections (6 μm). Paraffin sections were incubated with Rabbit Anti-DSPP antibody (1:500, bs-10316R, Bioss, China) and Rabbit Anti-DMP1 antibody (1:500, bs-12359R, Bioss, China) primary antibodies at 4 °C overnight followed by secondary antibody incubation the next day.

### Statistical analysis

The differences between groups were tested using the student’s *t* test and the differences among multiple groups were tested using one-way ANOVA, *P* < 0.05 was considered statistically significant.

## Supplementary information

SUPPLEMENTAL MATERIAL

## Data Availability

The data that support the findings of this study are available from the corresponding author upon reasonable request.
