# Supplementary material for: Mandible-derived extracellular vesicles regulate early tooth development in miniature swine via targeting KDM2B
Source: Int J Oral Sci. 2025 Apr 27;17:36. doi: 10.1038/s41368-025-00348-w (PMC12034755; doi:10.1038/s41368-025-00348-w)
Supplement: Supplementary file 1 — SUPPLEMENTAL MATERIAL [file 41368_2025_348_MOESM1_ESM.docx]

**Mandible-derived Extracellular Vesicles Regulates Early Tooth Development in Miniature Swine via Targeting KDM2B**

Ye Li ^1#^, Meng Sun ^1, 2#^, Yi Ding^1, 2^, Ang Li^1, 2*^

^1^Key Laboratory of Shaanxi Province for Craniofacial Precision Medicine Research, College of Stomatology, Xi’an Jiaotong University, Xi’an, 710049, China.

^2^ Department of Periodontology, College of Stomatology, Xi’an Jiaotong University, Xi’an, Shaanxi, 710000, China.

*Corresponding author: Ang Li. Email: [drliang@mail.xjtu.edu.cn](mailto:drliang@mail.xjtu.edu.cn) Tel: 86-29-87216030 Fax: 86-29-87216030

Key Laboratory of Shaanxi Province for Craniofacial Precision Medicine Research, College of Stomatology, Xi’an Jiaotong University, Xi Wu Lu No.98, Xi’an, Shaanxi, P. R. China.

#Authors contributed equally to the article

Ye Li. Email: liye0309@mail.xjtu.edu.cn

Meng Sun. Email: sunmeng0207@stu.xjtu.edu.cn

Yi Ding. Email: doctording1@stu.xjtu.edu.cn

**Running title:** **Mandible-EVs Regulates Early Tooth Development**

**Supplementary Figures**


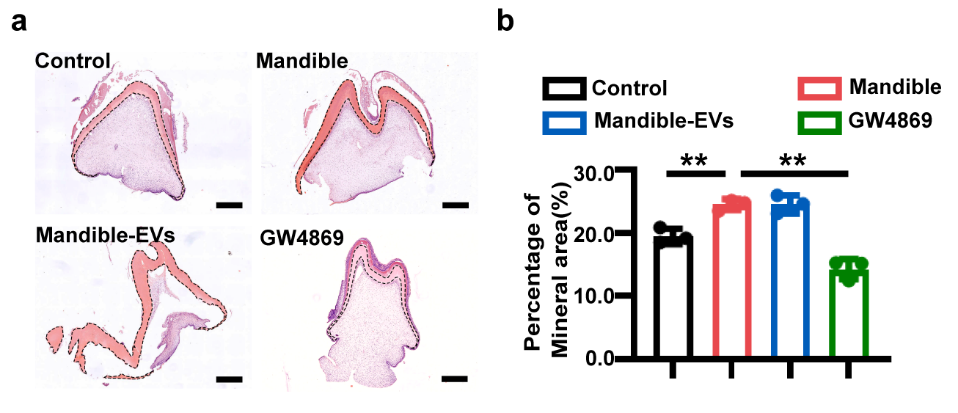


**Fig.S1 Mandible-EVs regulated tooth morphogenesis and mineralization.**

**a** H&E staining of tooth. Scale bar: 1 mm. **b** Quantitative data of HE. ** *P* < 0.01; n=3.


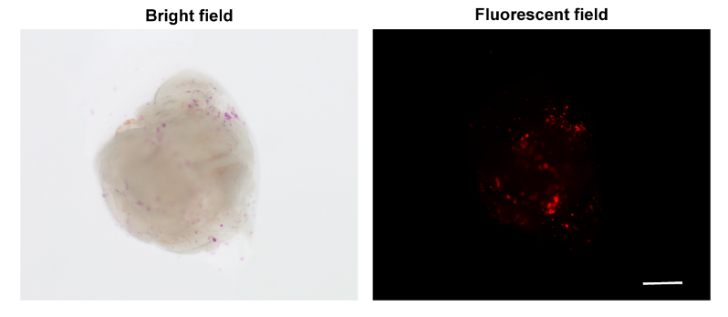


**Fig.S2 Mandible-EVs was internalized by tooth germ.** Mandible-EVs uptake by tooth germ were visualized by fluorescence stereomicroscope. Scale bar: 1 mm.


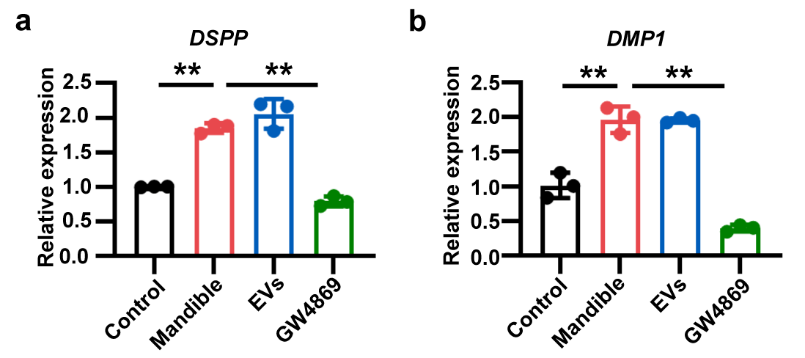


**Fig.S3 Mandible-EVs promoted odontogenic differentiation of dental mesenchymal cells. a** Real-time PCR assay of *DSPP* mRNAs in dental mesenchymal cells. **b** Real-time PCR assay of *DMP1* mRNAs in dental mesenchymal cells.** *P* < 0.01.


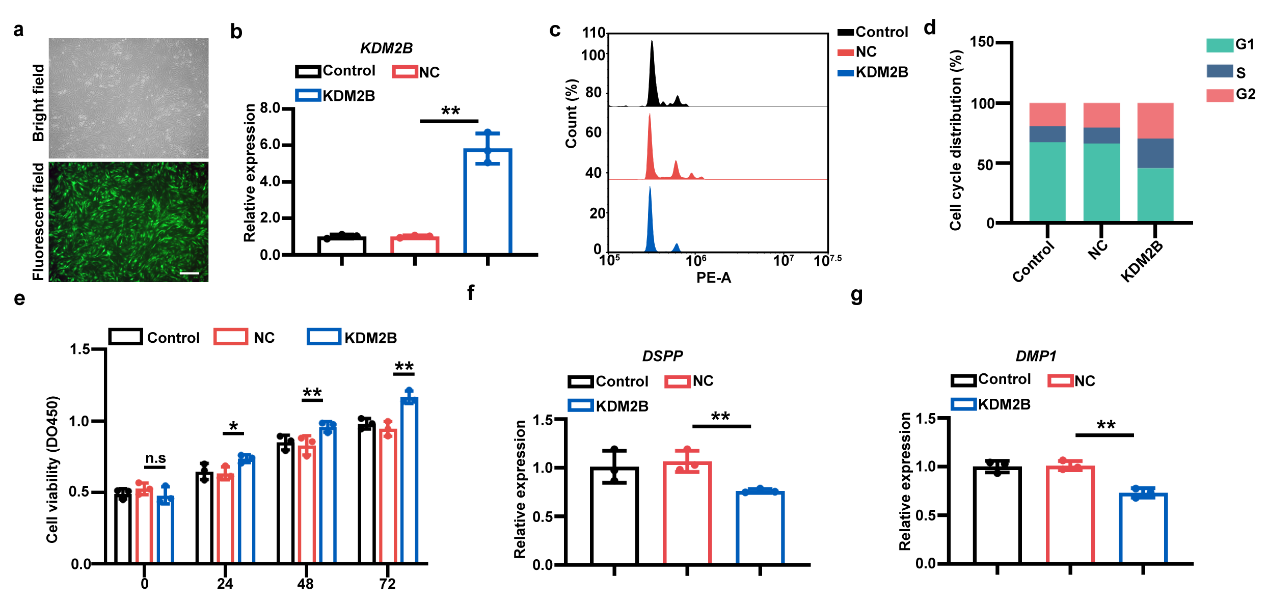


**Fig.S4 KDM2B promoted proliferation and inhibited odontogenic differentiation of dental mesenchymal cells.** **a** KDM2B overexpression dental mesenchymal cells were constructed. Scale bar: 100 μm. **b** The efficiency of KDM2B overexpression. **c** To identify the effect of KDM2B on cell cycle distribution, FCM was performed. **d** Percentage of G1, S, and G2 cell populations in the cycle phase based on FCM. **e** CCK-8 assay revealed the viability of dental mesenchymal cells at 0, 24, 48, or 72 h after KDM2B transfected. **f** The mRNA level of DSPP regulated by KDM2B. **g** The mRNA level of DMP1 regulated by KDM2B. n.s not significant, * *P* < 0.05, ** *P* < 0.01.


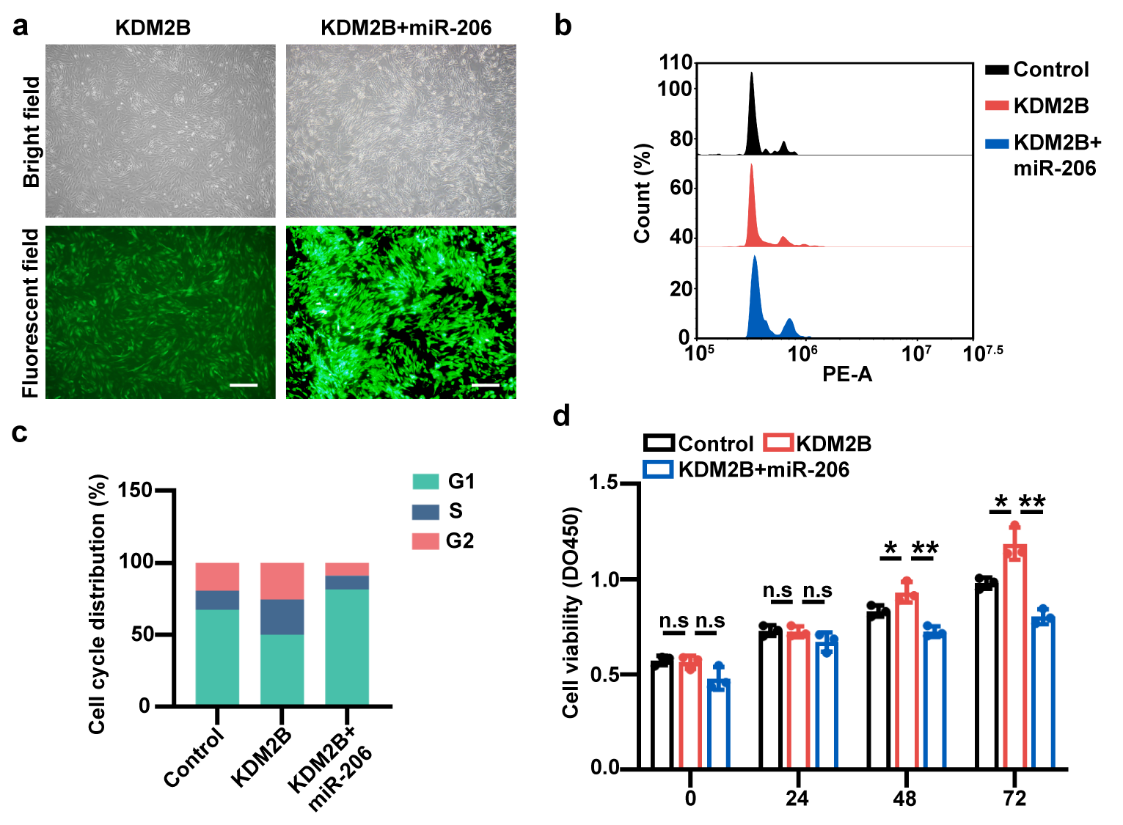


**Fig.S5 MiR-206/KDM2B regulate proliferation of dental mesenchymal cells. a** The efficiency of the miR-206 and KDM2B lentiviral vector. Scale bar: 100 μm. **b** To identify the effect of miR-206/KDM2B on cell cycle distribution, FCM was performed. **c** Percentage of G1, S, and G2 cell populations in the cycle phase based on FCM. **d** CCK-8 assay revealed the viability of dental mesenchymal cells at 0, 24, 48, or 72 h after KDM2B/miR-206 transfected. n.s not significant, * *P* < 0.05, ** *P* < 0.01.


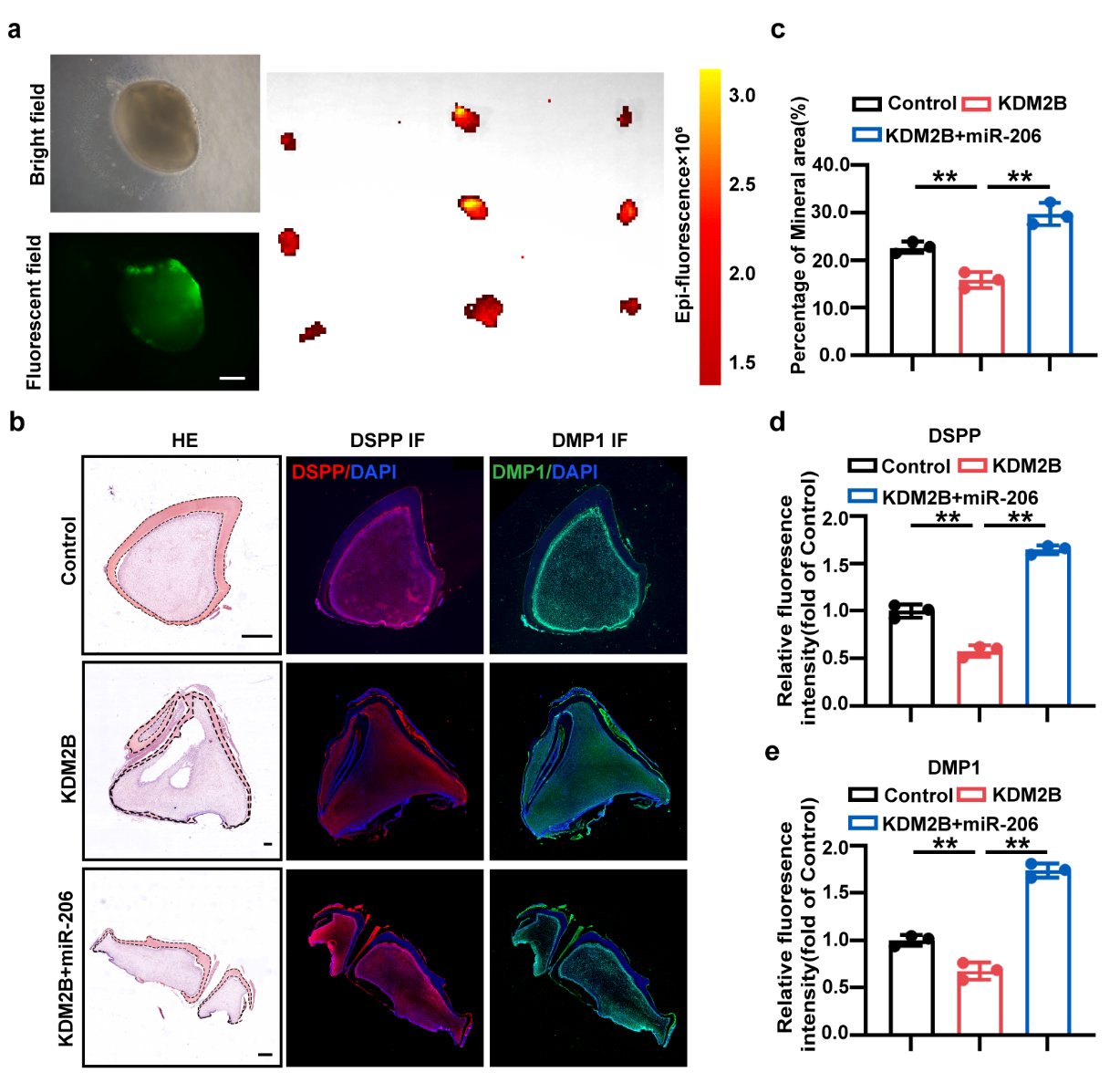


**Fig.S6 MiR-206/KDM2B regulated early tooth development.** **a** The efficiency of the miR-206 and KDM2B lentiviral vector. Scale bar: 100 μm. **b** H&E staining of tooth and DSPP, DMP1 expression in tooth regulated by miR-206/KDM2B. **c-e** Quantitative analysis. ** *P* < 0.01.

**Supplementary Materials and Methods**

*EVs characterization by atomic force microscopy (AFM)*

The 10 μl mandible-EVs diluted in PBS was aspirated onto the mica slices, air-dried, and observed by atomic force microscopy (Bruker, Multimode-8010800, USA). The data were processed by NanoScope Analysis software.

*EVs characterization by scanning electron microscopy (SEM)*

The mandible-EVs were fixed with 2.5% glutaraldehyde for 1 hour at RT, dehydrated with 30%, 50%, 70%, 90%, 95%, 100%, and 100% alcohol for 10 minutes each, and then dried under vacuum to make lyophilized powder. After the gold was sprayed onto the surface, the mandible-EVs were observed by scanning electron microscope (ZEISS, GeminiSEM 500, Germany).

*Raman Spectral data acquisition*

The mandible-EVs were analyzed by Raman microspectroscopy (Renishaw, inVia Qontor, UK). 10 µl drops of mandible-EVs suspension were deposited on aluminum paper. All of the measurements were performed at 20 mW and 532 nm through a 50× objective lens (NA 0.7). The acquisition time was 10 s per single take.

*Mandible-EVs analysis by 1H NMR*

After sonicated at 4℃ for 30 min, the mandible-EVs were centrifuged at 10,000 g for 10 min at 4℃. The precipitate was discarded and the supernatant was retained, concentrated and lyophilized. Then the powder was dissolved in 500 μl ddH2O and sonicated for 20 minutes. The supernatant was further transferred to an NMR cryotube, and analyzed by 1H NMR spectroscopy using an NMR spectrometer (BRUKER, AVANCE III HD 600 MHz, USA). MestReNova V14.0 software was used to process the data.

*Mandible-EV labelling and internalization assays*

Mandible-EVs were labeled with 1μM CM-DiI (MedChemExpress, Code No. 180854-97-1, USA) according to the manufacturer's instructions. Then E40 tooth germs were incubated with DiI-labeled mandible-EVs for 4h. Subsequently, tooth germs were washed with PBS and further fixed with 4% paraformaldehyde at room temperature for 20 minutes. After fixation, confocal fluorescence imaging was done on a fluorescence stereomicroscope (Zeiss, AXIO Zoom.V16, Germany).

*Real-Time PCR*

Total RNA was extracted using AG RNAex Pro Reagent (Code. 21101, Accurate Biology, China). Reverse transcription was performed by miRNA 1st strand cDNA synthesis kit (Code No. AG11716, Accurate Biology, China). The reverse-transcribed cDNA was further analyzed by SYBR® Green Premix Pro Taq HS qPCR Kit Ⅱ (Code. AG11702, Accurate Biology, China).

**Table1** Primer sequences for RT-qPCR.

| **Gene** | **Assay** | | **Forward (5’-3’)** | **Reverse (5’-3’)** |
| --- | --- | --- | --- | --- |
| KDM2B | | RT-qPCR | CCCTGCGGCTCATCATTC | AGGAGTCTCGGGTGGTGGT |
| DSPP | | RT-qPCR | GGAGGACGACGACCATAGC | CTCACTCTTGGAGGTGTTGTCT |
| DMP1 | | RT-qPCR | AGGTGAAAGCCGATCAGTGG | GCTTCTGCGGAGTCACTCTT |
| GAPDH | | RT-qPCR | AGGGCTGCTTTTAACTCTGGC | CGTGGGTGGAATCATACTGGA |
| ssc-miR-206 | | RT-qPCR | GCGCGTGGAATGTAAGGAAGT | AGTGCAGGGTCCGAGGTATT |
| U6 | | RT-qPCR | GGAACGATACAGAGAAGATTAGC | TGGAACGCTTCACGAATTTGCG |
